# Supplementary material for: Early alpha/beta oscillations reflect the formation of face-related expectations in the brain
Source: PLoS One. 2021 Jul 26;16(7):e0255116. doi: 10.1371/journal.pone.0255116 (PMC8312971; doi:10.1371/journal.pone.0255116)
Supplement: S1 File — Repeated measures cluster permutation test approach. (PDF) [file pone.0255116.s001.pdf]

S1 File

Alternative ERP analysis

The N170 amplitude was quantified by measuring the mean amplitude within the timeframe of 150 – 200ms in relation to a pre-stimulus baseline of 200ms. For this computation, the Mass Univariate ERP Toolbox for MATLAB [1] was employed to analyse the mean amplitude across selected channels (TP7/TP8, P7/P8) and within the respective timeframe. The ERPs for expected and random images were subjected to repeated measures cluster permutation tests with 5000 random iterations to correct for multiple comparisons. In line with our hypothesis that expected faces elicit a modulation in the N170 amplitude in comparison to random faces, a dependent, one-tailed *t*-test was computed for each electrode and timepoint (a total of 100 comparisons). Clusters arose when the one-tailed *t*-tests resulted in a *p*-value of or less than .05. The sum of the *t*-values within each cluster were compared and the maximum values extracted throughout each permutation to derive the null hypothesis distribution. Our test statistic of interest was thus deemed significant, if it grounded outwith the 95% tail of the derived distribution.

A significant reduction in amplitude was observed for expected in comparison to random images ($p = .039$) at left parietal and temporal-parietal channels (Fig 2A). The cluster permutation test also conveyed that this positive, left-lateralised cluster extends from approximately 158 – 188ms peri-stimulus (Fig 2A). In contrast, no significantly altered amplitude for expected faces ($p = .172$) was evident across the equivalent right-lateralised channels (Fig 2B).

References

1. Groppe DM, Urbach TP, Kutas M. Mass univariate analysis of event-related brain potentials/fields I: A critical tutorial review. Psychophysiology. 2011;48(12): 1711–25. doi: 10.1111/j.1469-8986.2011.01273.x
