# Supplementary material for: Early alpha/beta oscillations reflect the formation of face-related expectations in the brain
Source: PLoS One. 2021 Jul 26;16(7):e0255116. doi: 10.1371/journal.pone.0255116 (PMC8312971; doi:10.1371/journal.pone.0255116)
Supplement: Attachment — Submitted filename: Review Report_Roehe_Plos_One_May_2021.docx [file pone.0255116.s002.docx]

**Review Report**

**Early alpha/beta oscillations reflect the gradual formation of face-related expectations in the brain**

This is a particularly well designed and interesting study by Roche and colleagues to understand the origination and development implicit expectation using EEG. Recent evidence further suggests that both pre-stimulus neuronal oscillations and peri-stimulus event-related potentials are reliable biomarkers of implicit expectations arising from statistical learning paradigms. Employing such a paradigm in gender-classification task based on face stimuli they investigated attenuation of ERP signals, Pre-stimulus Alpha/Beta oscillations and Post-stimulus GBA determine the temporal constraints of face-related expectation formation. While I found the research questions are all relevant and methods employed here are technically accurate and the overall narrative also presented well, I do have the following questions, suggestions and comments.

**Introduction**

\# In my opinion, a more challenging issue I find for all these perceptual paradigms is how much perception of familiar or surprising occurrences are devoid of memory processing. There has to some involvement of component of memory specifically when the participants get trained in what kind of occurrences are expected versus not. Due to this training, they form a representation in memory of those perceptual prototype. Hence, I wonder how much of the face related expectation is aided by the service of memory and if you could dissociate what neurophysiological correlates are driven particularly by perceptual prediction and expectation versus memory component. Indeed you write about habituation and memory effects and repetition suppression effects in the discussion section.

\# One of the major issues of this work that I find is I am not too sure if I completely understand how the broader question asked and the overarching goal and hypothesis of this study design is any different from what has been investigated in some of the earlier works to address the following,

*How low-frequency neuronal oscillations act as carriers of sensory evidence and top-down predictions, respectively (von Stein and Sarnthein 2000; Bastos et al. 2012). In other words, whether slow pre stimulus alpha oscillations in task-relevant brain regions are stronger in the presence of predictions, whether they influence early categorization processes, and whether this interplay indeed boosts perception in general.*

https://pubmed.ncbi.nlm.nih.gov/26142463/

It would be crucial to delineate what separates out this study from the previous ones to be able to appreciate more the importance and exclusiveness of the findings.

\# In lines 76-78, authors write that “*The insight that the projection of prediction errors is mediated by high gamma frequency (60 – 100Hz) synchronisation, which requires a greater energetic cost than lower frequencies [8,9]”,* This statement is understandable but how does that shines light on the underlying processes shaping variability of ERPs is not clear. Perhaps, you could consider revising this statement.

\# Also, please explain why lesser Cognitive resource would suggest diminished ERPs. Is there already worked out inverse relationship between no. of Cognitive Resource and amplitude/power of ERP response or *vice versa*. Could please cite appropriate reference to justify this relationship?

\# The opposite such as amplified ERPs are evident for arbitrary or surprising occurrences. This is of course similar to what you expect for familiar versus unfamiliar stimuli or novel stimuli which is not consolidated yet in the memory. However, those which are already consolidated via prior experience/learning are already very familiar (e.g., expected faces) and should give similar to repetition suppression effect (repeated exposure). similarly, for surprising faces one should expect very similar effect as oddball stimuli. I would appreciate if you could you provide some of your reasoning against this comment?

\# Authors write “*Drawing upon this, it seems apparent that somewhat unexpected events are predominantly distinguishable from expected occurrences by enhanced gamma-band activity (GBA).”* There could be multiple interpretation of enhanced gamma-band activity. One specific reason you mention is supported by few recent studies which suggest that enhanced GBA could be involved in encoding and updating internal representation. However, thinking loud along the line of reasoning you have outlined in the Introduction I wonder whether this also has anything to do with deployment of larger number of cognitive resources while processing somewhat unexpected events. If you could kindly comment on this.

**Materials and methods**

\# You do not have a gender balanced sample for this study. I am wondering whether that has any impact on the gradual built up of implicit expectation and top-down prediction of expected face occurrences. Previous studies have shown that in the primary visual cortex while decoding faces and expressions, women showed a more bilateral functioning than men and there are some gender differences while processing neutral and affective faces. There are also difference that are found in the EEG N1 response as well as during oddball N170 amplitude response. Could you please provide your reasoning?

\# The authors hypotheses regarding the enhancement Alpha/Beta power pre-stimulus as top down prediction of expected faces. I am wondering why hypothesize about amplitude/power and why not phase of pre stimulus Alpha/Beta. I would imagine phase of pre stimulus Alpha may also play a crucial role. Also, this change in pre stimulus Alpha power could just reflect the ongoing background excitability which has a biophysical basis and has got nothing to do with any prediction about the faces. Please comment on this.

\# In lines 214-215, authors have mentioned about the selection of pre-post durations, however, not provided any details with regards to what is the basis of this selection.

The authors write, “-*200ms pre- to 600ms post-stimulus onset for ERPs. The 200ms prior to stimulus onset served as a baseline.* *For the TFA, data was epoched from -2000ms to approx. 1500ms, time-locked to stimulus onset.”*

Any comment on what exactly guided your selection of a specific pre stimulus duration as we know from previous studies that the choice of pre stimulus duration can have significant impact on the outcome of power changes and involvement of specific frequency band of interest. Hence, what frequency bands subserve the prediction process is very much sensitive to the choice of those parameters e.g., pre stimulus duration.

**Results**

\# Kindly explain why a title as ***“****Early alpha/beta oscillations reflect the gradual formation of face-related expectations in the brain” deemed appropriate.*

Is it really gradual over the entire period of pre stimulus duration or is it an accelerated process just before the onset of the expected image? It seems to be the latter based on the following results unless I did not follow these results correctly.

A significant positive relationship was, however, observed between the modulation of alpha/beta power underlying the final peak (8-30Hz; 2500 – 3000ms) and the modulation of the N170 (Spearman’s rho = .46, p = .021, 95% 381 CI [0.14 0.69]; Fig 5).

**Discussion**

I enjoyed reading the discussion section. However, I could not resist myself repeating my last comment again. Discussion section actually begins with what was my last comment for the previous section. It seems to me a more appropriate title hiding in the statement below,

“Predictable visual events are often met by implicit expectations to allow the brain to reserve cognitive resources.” Authors should certainly clarify which one of the two is more appropriate interpretation here the gradual development and built up of predictions prior to expected stimulus onset or an accelerated process showing tendency towards conserving cognitive resources in the process of predictions of expected outcome.

\# In lines 426-427 I suggest please provide some relevant references when you are discussing right lateralized habituation effects.

“These habituation effects or memory-driven modulations were found to be predominantly right-lateralised.” (reference please)

Is this memory-driven modulations of N170 ERPs predominantly right-lateralised irrespective of gender in face related expectation. Related to my earlier question about selected sampling.

\# I think the authors cited and covered references which are most relevant to their findings based on ERPs, pre stimulus brain activity, GBA.

However, I would like to point out that the recent studies suggest frequently reported negative relationship between pre-stimulus α/Beta power and stimulus detection or behavioural performance may be explained by changes in detection criterion. I am wondering whether there is something similar is happing here for example Pre stimulus power change in Alpha/Beta is driven by the state of neural excitability (brain states of individual participants), rather than top-down prediction of expected occurrences. Could you please comment on this. Related to my earlier question on background excitability.

\# The authors write in line 512 One could question why this pre-stimulus enhancement in alpha/beta power fluctuates instead of being a stable and continual increase in power leading right up to the presentation of expected targets.

I do have the same question and also related to my previous question of gradual bult up of expectation versus final peak increase before the presentation of expected targets. You have already provided an elaborate and solid reasoning in lines 515-521. But still curious whether this transient enhancement of power in the Alpha/Beta band should be considered at all for the entire inter-stimulus duration of 3 seconds as you have mentioned is too long a duration. Somehow, It seems these individual peaks are fundamentally functionally distinct and more meaningful pre stimulus anticipatory power change is occurring over a relatively short-interval of time frames with regards to significant functional attenuation the amplitude of ERPs to the presentation of expected targets. Hence, the clear role of these three peaks at least the first two and in particular, the middle one is not so clear after all. The waxing and waning of pre stimulus Amplitude/power change over certain time intervals and windows could be purely based on the change in background excitability and brain states.

I could also anticipate substantial inter individual and inter-trial differences in the processes of how expectations reflected in increased alpha/beta activity, which makes processing of expected stimuli more efficient for some participants and consequently reduces the amplitude of the N170 but not necessarily with the same efficiency and reduction for others.
